# The Relationship between Scapular Upward Rotation and Shoulder Internal and External Rotation Isokinetic Strength in Professional Baseball Pitchers

**DOI:** 10.3390/healthcare9060759

**Published:** 2021-06-18

**Authors:** Byung Gon Kim, Seung Kil Lim, Sunga Kong

**Affiliations:** 1 QOLFIT Training Center, Seoul 04794, Korea; atckbg@gmail.com; 2 Department of Exercise Prescription, Dongshin University, Naju 58245, Korea; es007-0@hanmail.net; 3 Department of Clinical Research Design and Evaluation, SAIHST, Sungkyunkwan University, Seoul 06351, Korea; 4 Patient-Centered Outcomes Research Institute, Samsung Medical Center, Seoul 06351, Korea

**Keywords:** scapular upward rotation, humerus elevation angle, isokinetic strength, baseball

## Abstract

This study aims to assess the relationship between scapular upward rotation (SUR) across varying humeral-elevation angles (HEAs) and shoulder isokinetic strength and ratio in professional baseball pitchers. The subjects were professional baseball pitchers (*n* = 16) without a history of shoulder injury in the last six months. The subject’s SUR angles were measured with the humerus elevated at HEAs of 0° (at rest), 60°, 90°, and 120° to the scapular plane. Shoulder isokinetic strength was evaluated for shoulder internal rotation (IR) and external rotation (ER) strength (PT%BW and TW%BW), and the ER/IR strength ratios were determined at 60, 120 and 180°/s using an isokinetic dynamometer. The SUR angle at an HEA of 0° was positively correlated with IR strength at 120°/s (r = 0.535) and 180°/s (r = 0.522). The SUR angle at an HEA of 60° was negatively correlated with the ER/IR strength ratios at 60°/s (r = −0.505) and 120°/s (r = −0.500). The SUR angle at an HEA of 90° was negatively correlated with the ER/IR strength ratios at 60°/s (r = −0.574; r = −0.554) and 120°/s (r = −0.521; r = −0.589) as well as with ER strength at 180°/s (r = −0.591, r = −0.556). The SUR angle at an HEA of 120° was negatively correlated with ER strength at 60°/s (r = −0.558), 120°/s (r = −0.504; r = −0.524), and 180°/s (r = −0.543) and the ER/IR strength ratio at 60°/s (r = −0.517). In this study, we found that the ratio of isokinetic strength between ER and IR became closer to the normal range on increasing the SUR angle. In particular, an HEA of 90°, which resembles the pitching motion, showed a clear relationship between SUR, shoulder ER, and the ratio of ER/IR isokinetic strength in professional baseball pitchers.

## 1. Introduction

The scapula plays an important role in normal shoulder function [1]; it moves in coordination with the moving humerus [2,3]. Scapular humeral rhythm (SHR) is controlled within a physiological pattern throughout the full range of shoulder motion [4]. When the normal scapular kinematics that comprise SHR are altered, scapular dyskinesis occurs. Scapular dyskinesis is a nonspecific response to a host of proximal and distal shoulder injuries. Therefore, abnormal scapular movements are linked to shoulder problems [5,6].

Many previous studies on baseball players have demonstrated that repetitive movement of pitching leads to SHR changes and, occasionally, incomplete restoration of normal shoulder biomechanics [7]. A change in the scapular upward rotation (SUR) occurs after acute fatigue [8]. The throwing shoulder exhibits significant differences in scapular stability and glenohumeral mobility compared with the nonthrowing shoulder [9,10]. In particular, patients with glenohumeral shoulder instability are shown to have decreased SUR [11,12]. The alteration and instability of the scapula have been identified as problems with the activity of the shoulder girdle muscle in patients with impingement [13]. 

Previous SHR studies based on normal and abnormal shoulder movements include electromyography (EMG) studies [14,15] (to determine the role of muscles such as the trapezius and middle and lower serratus anterior, which can rotate and stabilize the scapula), cadaveric studies [16] (to understand the anatomical alignment of muscle and motion in patients with nerve injuries), and biomechanical modeling (to predict arm movement and muscle function) [13]. Furthermore, knowledge of muscle role and dysfunction in shoulder-related pathology is essential to provide potential guidance for interventions to improve shoulder movement and function. However, it is important to note that EMG activation of the muscles during exercise does not clarify whether the muscles are active in stabilizing, translating, or rotating roles without additional consideration of the biomechanics of movement and the joint complexes [17]. However, data regarding scapular stability and glenohumeral mobility in professional athletes are lacking. 

It is necessary to confirm the relationship between scapular movement and shoulder muscle strength in baseball pitchers for training programs and injury prevention [18]. However, to date, most SHR studies among baseball players have focused only on SHR-related muscle activation [19] and alterations in scapular kinematics [9,10]. Most of the previous studies were on injured patients. Therefore, there is no clear understanding of the relationship between SHR, such as SUR, and shoulder strength in active professional baseball pitchers without injuries [20].

In this study, the relationship between SUR and Shoulder IR and ER isokinetic strength was investigated in a retrospective design, based on the preseason of injury-free professional baseball pitchers.

## 2. Materials and Methods

### 2.1. Subjects

This study analyzed preseason measurement data from 16 pitchers of a Korean professional baseball A-team. The subjects were athletes with no history of shoulder injury in the last six months. The purpose of this study was fully explained before the experiment to all eligible players, and only those who gave consent to participate in this study were included in the measurements. We analyzed only the data (*n* = 16) for which the values of all measurement outcomes were valid. This study also presents the results of an additional data analysis from a previous study [21]. This retrospective study was approved by the Institutional Review Board of Sungkyunkwan University (SKKU-2020-11-026). In addition, sufficient power was achieved with a sample size of 16 or more by setting the effect size to 0.65, α = 0.05 and power = 0.80 through the G*POWER program. The characteristics of the subjects are presented in Table 1.

### 2.2. Measurement

To measure SHR, SUR was examined across a range of HEAs (0, 60, 90 and 120°) using a digital inclinometer (SPI-Tronic, Garden Grove, CA, USA). To evaluate the shoulder internal rotation (IR) and external rotation (ER), isokinetic strength and ratio were measured using an isokinetic dynamometer (CSMi Humac Co., Stoughton, MA, USA). The measurement method was as follows: 

#### 2.2.1. Scapular Upward Rotation

The SUR was measured at the spinal surface of the scapula using a digital inclinometer when the humerus was elevated. The digital inclinometer measurements were highly correlated with those of the modified electric inclinometer (r = 0.996); the intra-correlation coefficient was >0.892 [22]. The digital inclinometer was aligned to the center of the humerus and made an angle vertical to the humerus. The locator rods were Y-shaped and designed to rest comfortably over the bony contours of the scapula [23].

**Figure 1:**
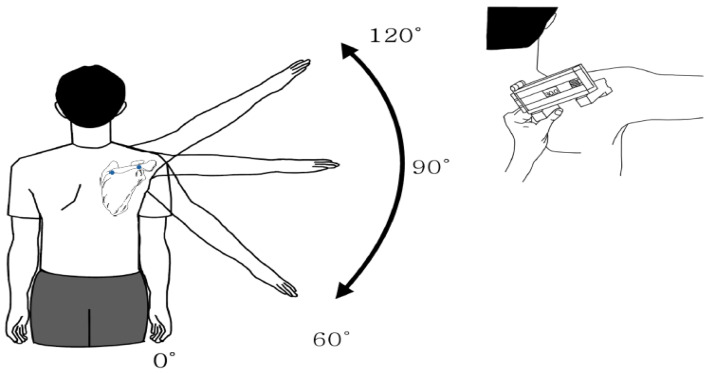
Measurement of scapular upward rotation (°) by humeral elevation.

The investigators assessed the angle of SUR in a resting position (HEA = 0°) and in the scapular plane at 60, 90 and 120° HEAs [24]. The HEAs were determined before scapular measurement by aligning the inclinometer along the long axis of the upper arm (Figure 1). The subjects elevated their arm in the scapular plane, using the wall as a guide against the dorsal surface of the hand, with the thumb positioned toward the ceiling throughout the testing procedure. The starting position began with the test arm in the resting position (HEA = 0°, arm at the side of the body) and the subject looked forward. The subjects elevated their arms until they reached the specific HEA (60, 90 and 120°). 

**Figure 2:**
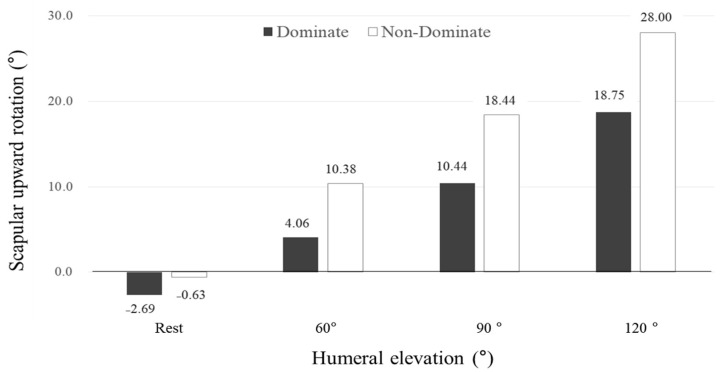
Scapular upward rotation (°) according to humeral elevation (°) in this study population.

At each angle, the subject was instructed to hold that position while the digital inclinometer was placed over the scapula and SUR was measured. The locator rods were positioned over the posterolateral acromion and root of the scapular spine (Figure 1). The subject’s hand returned to the resting position after each measurement to minimize fatigue. Rest time of approximately 20–60 s was maintained [10]. The characteristics of the subjects are presented in Table 2. In this study population, the SUR angle across a range of HEAs was lower in the dominant arm than in the nondominant arm. For humeral elevation at rest and HEAs of 60, 90 and 120°, the SUR angles of the nondominant arm were −0.6, 10.4, 18.4 and 28.0°, respectively, whereas the SUR angles of the dominant arm were −2.7, 4.1, 10.4 and 18.8°, respectively (Figure 2). 

#### 2.2.2. Shoulder Isokinetic Strength

Shoulder IR and ER isokinetic strength and ratio were measured using the isokinetic equipment CSMi (HUMAC Co., Stoughton, MA, USA). Measurements were performed three times at 60, 120 and 180°/s. [25]. Subjects were in the supine position during measurements, the shoulder joint was abducted by 90° and the elbow was flexed by 90°, and then the handle was held. In addition, for the objective evaluation of the total work done, the range of motion (ROM) of the joint was fixed at 30° of IR and 90° of ER. This position was chosen based on the specificity of muscle function and joint position angles with respect to the throwing motion, because it is similar to the elevation angle used during throwing motion [26]. The ICC of the isokinetic device ranged from 0.69 to 0.92 [27]. The dynamometer input shaft was aligned with the axis of rotation of the glenohumeral joint. The torso and forearm were fixed with a belt to minimize force action on body parts other than the shoulder. To increase the reliability of the examination, training was performed such that the pelvis and scapula and shoulders did not fall off the equipment table [28]. Isokinetic strength data were recorded during five maximal repetitions of IR and ER. The isokinetic strength was calculated as peak torque percentage normalized to body weight (PT/BW × 100, PT%BW, %) during IR and ER, and the ratio between the two (ER/IR × 100, %). Thereafter, the total work per body weight (TW/BW × 100, TW%BW, %) during IR and ER was measured, and the ratio (ER/IR × 100, %) was calculated. 

### 2.3. Statistical Analyses

All variables were measured using STATA 15.0 (Stata Corp LP, College Station, TX, USA) and are presented as means and standard deviations. We confirmed the normality of the small data sizes with a Wilk-Shapiro test. Pearson’s correlation was performed to determine the relationship between the SUR angle and shoulder isokinetic strength at each angular velocity. In addition, the relationship between the SUR angle and isokinetic strength ratio at an HEA of 90°, which resembles the arm cocking phase during throwing, was analyzed using linear regression. The level of statistical significance was set at α = 0.05.

## 3. Results

This study was conducted on professional baseball pitchers (*n* = 16) with an average career duration of 13 years. The SUR angles of the dominant arm were −2.7, 4.1, 10.4 and 18.8°, respectively (Figure 2). At 60, 120 and 180°/s, ER isokinetic strengths were relatively high compared to the IR in the dominant arm. Therefore, the ER/IR ratio was close to 1:1 (Table 1).

The SUR angle for humeral elevation at rest was moderate positively correlated with the isokinetic strength during IR at 120°/s PT%BW (r = 0.535 (95% CI: 0.053; 0.815); *p* = 0.033) and 180°/s PT%BW (r = 0.522 (95% CI: 0.036; 0.809); *p* = 0.038). However, it did not correlate with the isokinetic strength at ER and ratio between ER and IR (Table 2). 

The SUR angle at an HEA of 60° was moderate negatively correlated with the ER/IR isokinetic strength ratio at 60°/s PT%BW (r = −0.505 (95% CI: −0.800; −0.012); *p* = 0.046) and at 120°/s PT%BW (r = −0.500 (95% CI: −0.798; −0.005); *p* = 0.049) (Table 2).

The SUR angles at an HEA of 90° were moderate negatively correlated with the isokinetic strength during ER of 180°/s PT%BW (r = −0.591 (95% CI: −0.841; −0.135); *p* = 0.016). And, the SUR angles at an HEA of 90° were moderate negatively correlated with the ER/IR isokinetic strength ratio at 60°/s PT%BW (r = −0.574 (95% CI: −0.833; −0.110); *p* = 0.020), 60°/s TW%BW (r = −0.554 (95% CI: −0.824; −0.081); *p* = 0.026), 120°/s PT%BW (r = −0.521 (95% CI: −0.808; −0.034); *p* = 0.039), and 120°/s TW%BW (r = −0.589 (95% CI: −0.840; −0.132); *p* = 0.016), and 180°/s TW%BW (r = −0.556 (95% CI: −0.824; −0083); r = 0.025) (Table 2).

The SUR angles at an HEA of 120° were moderate negatively correlated with the isokinetic strength during ER of 60°/s PT%BW (r = −0.558 (95% CI: −0.825; −0.086); *p* = 0.023), 120°/s PT%BW (r = −0.504 (95% CI: −0.800; −0.011); *p* = 0.047), 120°/s TW%BW (r = −0.524 (95% CI: −0.809; −0.037); *p* = 0.037), and 180°/s PT%BW (r = −0.543 (95% CI: −0.818; −0.065); *p* = 0.030). The SUR angles were also moderate negatively correlated the ER/IR isokinetic strength ratio of 60°/s PT%BW (r = −0.517 (95% CI: −0.806; −0.029); *p* = 0.04) (Table 2).

**Figure 3:**
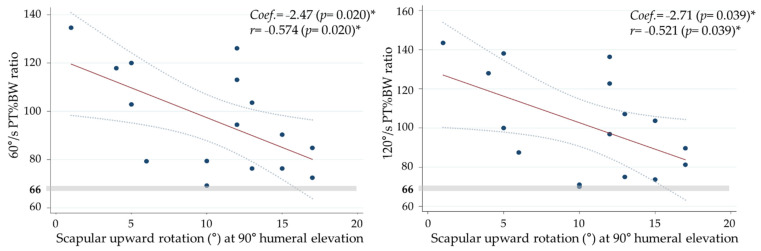
The relationship between scapular upward rotation (°) at 90° humeral elevation and ER/IR (PT%BW) ratio of 60°/s and 120°/s. (* *p* < 0.05).

A linear regression analysis showed that at an HEA of 90°, the ER/IR ratio at 60°/s PT%BW and 120°/s PT/BW decreased by −2.47 (95% CI: −4.481; −0.452) (*p* = 0.02) and −2.71 (95% CI: −5.251; −0.163); *p* = 0.039), respectively, when the SUR angle increased by 1°. As a result, the ER/IR ratio was close to a rate of approximately 66%. However, the ER/IR ratio at 180°/s PT%BW did not significantly decrease (r = 0.99, *p* = 0.339) (Figure 3).

## 4. Discussion

In this study, we found that the SUR angle at an HEA of 0° (at rest) was moderate positively correlated with shoulder isokinetic strength during IR, and the SUR angles at 90° and 120° HEAs were moderate negatively correlated with shoulder isokinetic strength during ER. In particular, the ratio of isokinetic strength between ER and IR became closer to the normal range on increasing the SUR angle at an HEA of 90°. These results demonstrate the relationship between the SUR angle and shoulder IR and ER isokinetic strength in professional baseball pitchers.

Our study population showed that the SUR angle, across a range of HEAs, was limited to the dominant arm compared with the nondominant arm among professional baseball pitchers. This is consistent with those of another study that shows that the SUR angles among pitchers are significantly lower at HEAs of 60° (3.9°) and 90° (4.4°) than those among position players [10]. In addition, previous studies reported that shoulder impingement syndrome [29] and limitations of subacromial clearance cause a 4–5° difference in scapular movement [30]. It is known that the ratio of upward rotation of the scapula to humeral elevation is 2:1 [31]; however, the SUR angles of the pitchers in our study were lower. Laudner et al. (2007) proposed three causes of SUR limitation in pitchers [10]. First, laxity and tension of the inferior glenohumeral ligament may be decreased, reducing support for SUR [32]. Second, muscle fatigue may be caused by repetitive pitching. Decreased activation and strength of the lower, middle and upper trapezius, as well as the serratus anterior muscles, have been shown to significantly limit the amount of SUR owing to shoulder fatigue among pitchers [8]. Finally, tightness of the downward rotators of the scapula, namely the rhomboids, levator scapula and pectoralis minor muscles, may limit the amount of upward rotation available. In addition, other studies have demonstrated that decreased SUR among pitchers may result in detrimental cavity/compression alterations between the humerus and glenoid [1]. Decreased SUR at an HEA of 90° might be related to the development of pain through mechanical compression of the rotator cuff [33]. Meanwhile, an increase in SUR in accordance with HEAs could be important for avoiding subacromial impingement [34]. In general, it has been known that the rotator cuff muscles primarily stabilize the scapula-humerus joint, but also contribute significantly to movement, such as IR and ER [35]. However, our study did not include patients with symptomatic rotator cuff tears but confirmed a decrease in SUR in professional baseball pitchers preparing for the season.

Our study confirmed that the SUR angle at rest was moderate positively correlated (+) with shoulder IR isokinetic strength. Previous studies showed that restricted SUR is linked to IR ROM limitation, such as glenohumeral internal rotation deficit [36,37]. In addition, the ROM and strength of IR in the throwing shoulder are limited due to osseous adaptation, posterior muscle tightness and posterior inferior capsule tightness [38]. This is also caused by weakness of the rotator muscle and variations in scapula-humeral rotations (dyskinesis) [39,40]. A previous study demonstrated that contracture of the posterior capsule altered normal glenohumeral kinematics [41]. If a pitcher has a posterior capsular contracture with decreased IR, it restricts the humerus from rotating externally into its normal posteroinferior position in the arm cocking phase of throwing [42]. Our study showed that an increase in SUR and an improvement in shoulder IR isokinetic strength were positively correlated with the resting position (HEA = 0°). This may be due to the inactivation of the scapular downward rotation muscle at the resting angle.

Furthermore, we found that the SUR angle was moderately negatively correlated (−) with shoulder ER isokinetic strength and shoulder ER/IR isokinetic strength ratio. An increase in the SUR angle brought the ER/IR isokinetic strength ratio closer to the normal range. In general, the ratio of shoulder ER/IR isokinetic strength is 2:3 or 66% [18,43]. The ratio of ER/IR isokinetic strength and agonist-antagonist strength is often used to identify risk factors of shoulder pathology [44]. This is because the imbalance in the ratio of ER/IR strength can cause problems with dynamic stabilization and increase injuries of the joint [45,46,47,48]. In addition, an imbalance in the strength ratio is linked to fatigue [49]. Muscle imbalances of the shoulder can be caused by unbalanced training regimens, training for a particular sport, work-related tasks and poor posture. Muscle imbalance can also occur secondary to other shoulder injuries. However, it remains difficult to distinguish adaptive and pathologic changes. Therefore, it is necessary to evaluate a pitcher’s movements during SUR and observe changes related to the scapula [4,50]. Our results indicate that managing SUR according to the HEA can not only ensure the mobility and stability of the scapula [50] but also prevent muscle damage to the shoulder joint [51,52].

Our results confirmed that the relationship between SUR limitation and rotation isokinetic strength is directly related to the arm cocking phase (HEA, 90° and 120°) during the throwing motion. Pitchers are shown to have significantly more passive external shoulder rotation at an HEA of 90° than position players [53]. In baseball players, the scapula must protract and rotate in the upward direction when the humerus is adducted horizontally during arm elevation in the arm cocking phase [54]. However, studies on muscle activation according to SUR mention that patients with symptomatic rotator cuff tears showed less SUR and higher activity of the levator scapulae on elevating the arm by 90° [34]. The minimum distance between the coracoacromial arch and rotator cuff insertion was observed to occur near or below an HEA of 90° [55]. Our results also showed that HEA of 90° and 120° were much more relevant than HEA 60°. Consequently, control of the scapular movement was confirmed to be linked with the muscle function of the shoulder joint during a pitcher’s throwing motion. Therefore, to solve the structural and functional problems of the shoulder joint and to prevent shoulder injury, SUR needs to be monitored. It is necessary to include the shoulder joint and scapula in a management program for performance improvement in players with repetitive throwing and overhead movements.

We confirmed the relationship between SUR across varying humeral-elevation angles (HEAs) and IR and ER shoulder isokinetic strength and ratio in professional baseball pitchers without injury. In our results, a decrease in SUR was associated with a decrease in shoulder IR and an increase in shoulder ER. Previous studies revealed strong evidence that reduced IR endurance and strength ratios are predictive of shoulder injury [56], and strength imbalances assessed by isokinetic dynamometry are linked to injury [57]. Numerous articles provide recommendations for the rehabilitation of strength imbalances to prevent injury [48,58]. Therefore, to monitor and evaluate the scapulohumeral rhythm periodically and to maintain SUR and shoulder IR and ER strength and ratio, both scapular and shoulder joint management must be included in the program for injury prevention and functional recovery among baseball pitchers.

This study had some limitations. First, as a retrospective study, our findings only confirmed the association between SUR and shoulder IR and ER isokinetic strength. However, the cause of restricted SUR remains unclear. By analyzing the relationship between SUR and shoulder isokinetic strength according to the HEA, we attempted to clarify the meaning of this relationship in context of the throwing motion of a professional baseball pitcher. Second, this study was cross-sectional and conducted during preseason; therefore, the effect of SUR restriction on the occurrence of shoulder injury was unknown. However, our findings provide evidence for designing shoulder joint and scapula-related training programs for pitchers. Third, the athlete’s career or age were unaccounted for, and the sample size was small. However, biomedical research on professional baseball pitchers is limited. Further studies are needed to develop a rehabilitation program and to prevent damage to athletes by revealing other structural and functional problems of the scapula and shoulder joints through multilateral analysis. 

## 5. Conclusions

In this study, we found that the SUR angle at rest was highly correlated with positive IR isokinetic strength, and the SUR angle at an HEA of 90° was moderately negatively correlated with ER and the ER/IR isokinetic strength ratio. In particular, an HEA of 90°, which resembles the pitching motion, showed a clear relationship between SUR, shoulder ER, and the ratio of ER/IR isokinetic strength in professional baseball pitchers. In the future, through a cohort study, it will be necessary to confirm whether the imbalance of muscle strength and ratio of SUR and shoulder IR and ER is directly related to injury.

## Figures and Tables

**Table 1:** Characteristics of the subjects.

Variables		Mean ± SD	Range
Age (year)		23.94 ± 4.71	18–33
Height (cm)		183.31 ± 4.47	174–192
Weight (kg)		87.69 ± 8.17	76–108
Body Mass Index, (kg/m^2^)		26.08 ± 1.98	23.4–29.3
Career (years)		13.33 ± 3.50	8–20
60°/s PT%BW (%)	IR	31.63 ± 5.78	23–40
	ER	29.5 ± 3.44	23–36
	ER/IR	96.30 ± 20.98	69.2–134.6
120°/s PT%BW (%)	IR	29.75 ± 6.76	21–40
	ER	28.88 ± 3.90	21–39
	ER/IR	101.53 ± 25.39	71–145
180°/s PT%BW (%)	IR	33.63 ± 6.02	23–44
	ER	28.81 ± 3.53	24–36
	ER/IR	88.19 ± 18.95	66–128

SD, standard deviation; PT%BW, peak torque % body weight (%); IR, internal rotation; ER, external rotation; ER/IR ratio (ER/IR × 100).

**Table 2:** The relationship between scapular upward rotation (°) and shoulder isokinetic strength.

Scapular upward Rotation Angle IR and ER Isokinetic Strength and Ratio		Humeral Elevation			
		Rest, r (*p*)	60°, r (*p*)	90°, r (*p*)	120°, r (*p*)
60°/s PT%BW (%)	IR	0.460 (0.073)	0.424 (0.102)	0.422 (0.104)	0.279 (0.296)
	ER	0.064 (0.814)	−0.392 (−0.133)	−0.418 (0.107)	−0.558 (0.023) *
	Ratio	−0.378 (0.149)	−0.505 (0.046) *	−0.574 (0.020) *	−0.517 (0.040) *
60°/s TW%BW (%)	IR	0.443 (0.086)	0.297 (0.265)	0.336 (0.203)	0.091 (0.738)
	ER	−0.012 (0.966)	−0.402 (0.123)	−0.457 (0.075)	−0.393 (0.132)
	Ratio	−0.399 (0.126)	−0.473 (0.064)	−0.554 (0.026) *	−0.318 (0.230)
120°/s PT%BW (%)	IR	0.535 (0.033)*	0.367 (0.163)	0.272 (0.308)	0.184 (0.495)
	ER	−0.045 (0.868)	−0.385 (0.141)	−0.435 (0.092)	−0.504 (0.047) *
	Ratio	−0.500 (0.051)	−0.500 (0.049) *	−0.521 (0.039) *	−0.448 (0.082)
120°/s TW%BW (%)	IR	0.311 (0.241)	0.243 (0.365)	0.309 (0.244)	−0.139 (0.607)
	ER	−0.027 (0.921)	−0.294 (0.269)	−0.277 (0.299)	−0.524 (0.037)*
	Ratio	−0.341 (0.197)	−0.470 (0.066)	−0.589 (0.016) *	−0.282 (0.290)
180°/s PT%BW (%)	IR	0.522 (0.038) *	0.005 (0.985)	−0.105 (0.698)	−0.088 (0.747)
	ER	0.071 (0.794)	−0.426 (0.100)	−0.591 (0.016) *	−0.543 (0.030) *
	Ratio	−0.368 (0.160)	−0.192 (0.476)	−0.256 (0.339)	−0.188 (0.485)
180°/s TW%BW (%)	IR	0.377 (0.150)	0.185 (0.493)	−0.132 (0.627)	−0.026 (0.923)
	ER	0.033 (0.903)	−0.333 (0.208)	−0.556 (0.025) *	−0.495 (0.051)
	Ratio	−0.418 (0.107)	−0.360 (0.171)	−0.264 (0.322)	−0.297 (0.264)

* *p* < 0.05; IR, internal rotation; ER, external rotation; ER/IR ratio (ER/IR × 100).

## Data Availability

Not applicable.
